# Reduction in squamous cell carcinomas in mouse skin by dietary zinc supplementation

**DOI:** 10.1002/cam4.768

**Published:** 2016-05-17

**Authors:** Jin Sun, Rulong Shen, Morgan S. Schrock, James Liu, Xueliang Pan, Donald Quimby, Nicola Zanesi, Teresa Druck, Louise Y. Fong, Kay Huebner

**Affiliations:** ^1^ Department of Molecular Virology, Immunology and Medical GeneticsThe Ohio State University Comprehensive Cancer CenterColumbusOhio; ^2^ Department of PathologyCollege of MedicineColumbusOhio; ^3^ Biomedical Sciences Graduate ProgramColumbusOhio; ^4^ Department of Biomedical InformaticsCenter for BiostatisticsThe Ohio State UniversityColumbusOhio; ^5^ Department of Pathology, Anatomy & Cell BiologySidney Kimmel Cancer CenterThomas Jefferson UniversityPhiladelphiaPennsylvania; ^6^ Present address: Department of PathologyMolecular PathologyThe Ohio State University Wexner Medical CenterColumbusOhio; ^7^ Present address: Beaumont‐Oakland University Medical SchoolGrosse PointeMichigan; ^8^ Present address: University of CincinnatiDepartment of Internal MedicineCincinnatiOhio

**Keywords:** DMBA‐induced skin tumors, Fhit knockout mice, tumor prevention, tumor suppressor‐deficient mice, zinc supplementation

## Abstract

Inadequate dietary Zn consumption increases susceptibility to esophageal and other cancers in humans and model organisms. Since Zn supplementation can prevent cancers in rodent squamous cell carcinoma (SCC) models, we were interested in determining if it could have a preventive effect in a rodent skin cancer model, as a preclinical basis for considering a role for Zn in prevention of human nonmelanoma skin cancers, the most frequent cancers in humans. We used the 7,12‐dimethyl benzanthracene carcinogen/phorbol myristate acetate tumor promoter treatment method to induce skin tumors in Zn‐sufficient wild‐type and Fhit (human or mouse protein) knockout mice. Fhit protein expression is lost in >50% of human cancers, including skin SCCs, and Fhit‐deficient mice show increased sensitivity to carcinogen induction of tumors. We hypothesized that: (1) the skin cancer burdens would be reduced by Zn supplementation; (2) *Fhit* ^−/−^(*Fhit,* murine fragile histidine triad gene) mice would show increased susceptibility to skin tumor induction versus wild‐type mice. 30 weeks after initiating treatment, the tumor burden was increased ~2‐fold in *Fhit* ^−/−^ versus wild‐type mice (16.2 versus 7.6 tumors, *P *<* *0.001); Zn supplementation significantly reduced tumor burdens in *Fhit* ^−/−^ mice (males and females combined, 16.2 unsupplemented versus 10.3 supplemented, *P *=* *0.001). Most importantly, the SCC burden was reduced after Zn supplementation in both strains and genders of mice, most significantly in the wild‐type males *(P *=* *0.035). Although the mechanism(s) of action of Zn supplementation in skin tumor prevention is not known in detail, the Zn‐supplemented tumors showed evidence of reduced DNA damage and some cohorts showed reduced inflammation scores. The results suggest that mild Zn supplementation should be tested for prevention of skin cancer in high‐risk human cohorts.

## Introduction

About 2 million people in the United States are diagnosed and treated for nonmelanoma skin cancer (NMSC) yearly, making it the most common form of cancer 1, and in 2012, there were nearly 100,000 cases registered in Great Britain 2, 3, 4, 5. The majority of NMSCs are basal cell carcinomas (BCC) or squamous cell carcinomas (SCCs) 6. Among humans, both BCC and SCC are more common in males than females, with a wider sex difference for SCC 6. SCC incidence in Great Britain increased by more than a third in males and females from 2002 to 2010, likely reflecting increased UV exposure 6. NMSCs have high cure rates due to visible precancerous lesions, treatment with various surgical methods and follow‐up histopathological analysis for confirmation of complete excision. Since most skin cancers occur on visible portions of the skin, the treatment methods can lead to considerable scarring of facial and other highly visible areas. Radiation therapy and topical chemotherapies are alternatives for more advanced cancers 7. Due to the curability of NMSC, it is generally not included in overall cancer statistics; however it can be fatal: ~2000 individuals die annually from NMSC in the U.S. alone. Although NMSCs metastasize infrequently, immunosuppressed patients or those on immunosuppressive medications (transplant patients) are at a 100‐fold higher risk of NMSC‐associated morbidity 8, with some patients developing hundreds of skin cancers. Thus, we sought to confirm a prevention method that would provide additional protection, particularly for fair‐skinned and immunosuppressed populations, a method that may prove efficacious for other cancers as well.

Zinc (Zn) is an essential trace element in man and animals, required for activity of ~2000 transcription factors and >300 enzymes 9. Dietary Zn deficiency has been linked to many human cancers, including esophageal SCC 10, head and neck 11, and digestive tract cancers 12. Likewise, studies of rodent models of chemically induced esophageal and forestomach cancers have shown that Zn deficiency results in significantly more tumors and more severe histopathological lesions 13, 14. Zn supplementation and replenishment in these Zn‐deficient animal models reversed tumor burdens and histopathological grade. In a recent study, we maintained Zn‐deficient and Zn‐sufficient mice on a regimen of Zn supplementation in the drinking water and found that even the Zn‐sufficient cohort benefited significantly from supplemental Zn during forestomach carcinogenesis, resulting in decreases in tumor number and severity of preneoplastic and neoplastic lesions 14.

We have also questioned whether Zn has antitumor efficacy in a Zn‐sufficient animal by investigating in Zn‐sufficient rats the efficacy of Zn supplementation on the progression of tongue SCC induced by exposure to 4‐nitroquinoline 1‐oxide in drinking water. Zn supplementation significantly reduced the incidence of papillomas and invasive carcinomas. The results demonstrated that Zn supplementation has chemopreventive efficacy against oral carcinogenesis in nutritionally complete animals, suggesting that Zn supplementation may be efficacious in the chemoprevention of human oral cancer 15.

Since Zn is an inexpensive supplement, readily available in developed countries, and could easily be included in human clinical trials, we set out to determine whether Zn supplementation would lower tumor burdens in a mouse NMSC preclinical model.

We chose to induce NMSC using the well‐characterized model for studying premalignant and malignant progression, the 7,12‐dimethyl benzanthracene/phorbol myristate acetate (DMBA/PMA) regimen 16. DMBA is oxidized by endogenous P450 enzymes to metabolites that form covalent DNA adducts. It is used to induce skin cancer in conjunction with PMA, a phorbol, which activates the signal transduction enzyme, protein kinase C, and promotes tumor development 17. We began these studies using C57BL/6J (B6) wild‐type (wt) mice that are naturally resistant to skin cancers (http://www.informatics.jax.org/mtbwi/tumorFrequencyGrid.do); we included (*Fhit,* murine fragile histidine triad gene) *Fhit* ^−*/*−^ mice on the B6 background, because Fhit (human or mouse protein) loss has been reported for skin SCCs 18, 19. We hypothesized that they would show enhanced sensitivity to induction of skin cancers versus the B6 strain. Fhit knockout mice have shown enhanced sensitivity to carcinogen induction of gastric and lung cancers 20, 21, 22, so we considered that this strain would provide a strong test of the Zn supplementation effect. Also, loss of expression of Fhit tumor suppressor protein occurs in >50% of human cancers 23. Fhit also serves as a genome caretaker preventing the onset of global genome instability 24, 25. Fhit‐deficient and haploinsufficient mice spontaneously develop lung tumors, lymphomas and liver hemangiomas at slightly higher incidences than wt mice 21 and 100% of Fhit knockout mice developed NMBA‐induced forestomach tumors, significantly more than wt mice 20, 21. We hypothesized that wt and Fhit‐deficient mice would develop increased numbers of induced NMSCs and that Zn supplementation would reduce tumor burdens.

## Materials and Methods

### Mouse strain, tumor induction, and Zn supplementation

**Figure 1:**
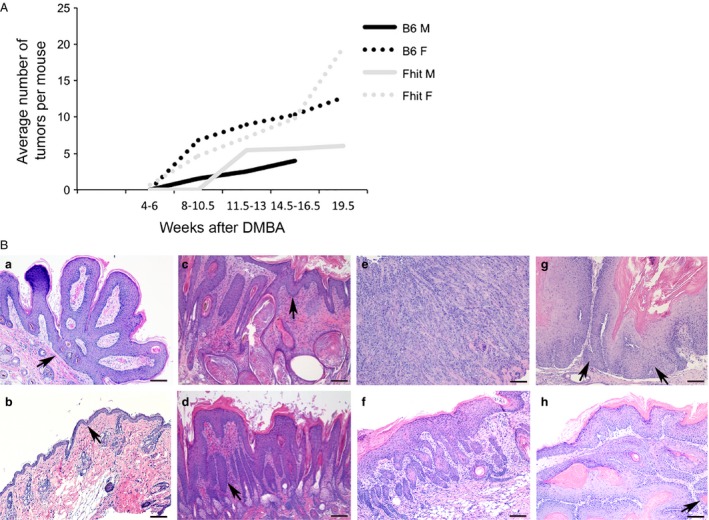
Timing of tumor induction and histopathology of DMBA/PMA‐induced skin tumors in mice with or without Zn supplementation. (A) Line graph showing the average number of tumors in wt and *Fhit* ^−*/*−^ male and female mice 4–19.5 weeks after treatment with DMBA. The data was from of a pilot study with 2–6 mice per group to estimate timing of tumor initiation in the mouse cohorts. (B) Representative photomicrographs of H&E stained skin lesions showing (a) a papilloma from a *Fhit* ^−*/*−^ female without Zn; (b) mild hyperplasia from a wt male with Zn treatment; (c) a severe hyperplasia from a *Fhit* ^−*/*−^ female with Zn treatment; (d) dysplasia from a *Fhit* ^−*/*−^ female with Zn treatment; (e) a poorly differentiated SCC with cords of highly atypical tumor cells diffusely infiltrating into the stroma and skeletal muscle from a *Fhit* ^−*/*−^ female without Zn; (f) a well‐differentiated SCC, with mild cytologic atypia from a wt female without Zn; (g) a moderately differentiated SCC, with enlarged tumor cells and prominent nucleoli, (single invasive tumor cell, left arrow; right arrow points to the tumor) from a wt male without Zn; (h) a well‐to‐moderately differentiated SCC (all portions in subcutaneous area are invasive) from a wt male with Zn treatment. Scale bar = 100 *μ*m. *Fhit,* murine fragile histidine triad gene; Fhit, human or mouse protein; H&E, hematoxylin & eosin; SCC, squamous cell carcinoma; PMA, phorbol myristate acetate.

Wild‐type B6 and *Fhit* ^−*/*−^ mice were examined for skin cancer susceptibility. B6 mice were from the Jackson Laboratory. *Fhit* ^−*/*−^ mice (~95% B6 genetic makeup) were generated as described 14, 26, backcrossed to B6 mice and carried as homozygous knockout mice; genotypes were confirmed before tumor induction. Mouse studies and experimentation were carried out according to protocols approved by the Ohio State University Institutional Animal Care and Use Committee. To induce tumors, the dorsal skin of 6–8‐week‐old mice was shaved to provide a 3 × 5 sq. cm area of skin for topical treatment with 50 *μ*g of DMBA (Sigma, St. Louis, MO) in 200 *μ*L acetone, followed by application of 7.5 *μ*g PMA (Sigma) 3 times/week for 30 weeks. A 20‐week pilot experiment was first performed on small cohorts using the same mouse strains and treatment protocol without Zn supplementation, to gage timing of tumor initiation in the different mouse strains and genders (see Fig. 1A for graph of tumor initiation/latency in the first 20 weeks, without Zn supplementation). For the 30 week experiment, each genotype included control and Zn‐supplemented cohorts with mouse groups ranging from 10 to 28 mice. Control mice received standard water ad libitum, whereas the treatment group received Zn‐gluconate (Life Extension, Ft. Lauderdale, FL) supplemented water (0.043 mg Zn/day/mouse, assuming ~7 mL water/day/mouse (equivalent to ~120 mg Zn/day for a human), following the initial DMBA treatment. All mice were fed standard lab rodent diet (Harlan Teklad 7912, irradiated rodent chow 7012). Mice were monitored weekly for tumor development and sacrificed after the final PMA treatment. Upon experiment termination, skin, liver, spleen, and kidney were grossly examined for tumors, which were not observed; skin tumors were measured and counted. The entire treated skin area was excised and cut into three sections that were fixed and processed for histology and immunohistochemistry (IHC) studies. Tumors (at least 2 mm in diameter) from individual skins with adjacent nontumor skin were isolated and separately processed for analysis.

To examine the role of inflammation in tumor burden reduction by Zn supplementation, we scored *Fhit* ^−*/*−^ and B6 mouse tumors for inflammation on a scale of 0–3 at the time of sacrifice.

### Histopathological analysis

Tissues were fixed in 10% buffered formalin for 2 days and then processed and embedded in paraffin. Skin sections were cut across all layers and stood on cut‐ends on slides. Slides were stained with hematoxylin and eosin (H&E) for pathological assessment, for enumeration of papillomas and carcinomas, or with specific antisera for IHC analyses. Skin lesions were graded and enumerated by pathologists (RS, JL) who were blinded to treatments. This analysis was performed by light microscopy using a Nikon Eclipse Ci microscope and photographs taken with a Nikon digital sight DSfi1 C camera. Skin tissue with two epithelial layers was designated normal, whereas mild and severe hyperplasia showed increasingly thickened epithelium. Squamous cell carcinomas were categorized based on three differentiation levels: poorly, moderately, and well‐differentiated.

### Immunohistochemical analysis

Based on previous studies of skin cancer induction and Zn supplementation, 8‐hydroxy‐2′ ‐deoxyguanosine (8‐OHdG), and *γ*H2AX were chosen for expression analysis in the various treatment groups 14, 27. Antisera used are listed in Table 1, with antigen retrieval and detection methods. IHC staining was performed in the histology/immunohistochemistry Core Lab of the Department of Veterinary Biosciences of The Ohio State University, by the following procedure: Skin tissue embedded in paraffin was cut at 4 *μ*m and placed on positively charged slides; slides were deparaffinized by xylene and rehydrated through graded ethanol solutions to water. For 8‐OHdG, and *γ*H2AX, antigen was retrieved by conditions listed in Table 1 followed by water washes. Slides were quenched for 10 min in a 3% hydrogen peroxide solution to block endogenous peroxidase and blocked with Dako serum‐free protein block (Dako North America, Carpinteria, CA) before 30 min incubation with primary antisera in Dako antibody at dilutions indicated in Table 1. After washes, slides were incubated 30 min with biotinylated secondary antisera. Slides were then incubated with Vector RTU ABC Elite (Vector laboratory, Burlingame, CA) complex for 30 min and were incubated with Dako DAB substrate chromogen for 5 min followed by counterstaining in hematoxylin for 15 sec. After dehydration through graded ethanol solution and xylene, slides were coverslipped.

**Table 1:** Primary antisera and detection methods for immunohistochemical assays

Antigen Retrieval	Primary antiserum	Source	Description	Dilution	Secondary Ab
Dako Proteinase K 15 min at RT	8‐OHdG	Millipore	AB5830, goat polyclonal	1:1000	Rabbit anti‐goat 1:200
Dako Target Retrieval Solution	*γ*‐H2AX	Bethyl	IHC‐00059	1:100	Goat anti‐rabbit 1:200
Dako Proteinase K 5 min at RT	MPO	Dako	A0398, rabbit polyclonal	1:400	Goat anti‐rabbit 1:1000

8‐OHdG, 8‐hydroxy‐2′‐deoxyguanosine; IHC, immunohistochemistry.

### Statistical analyses

To compare tumor burdens of mice with or without Zn supplementation, general linear models with fixed Zn treatment, gender, and their interactions were used to test overall effect of Zn supplementation for each mouse genotype. Poisson regression analysis was also conducted as sensitivity analysis to confirm the conclusions are robust for the counting data. Weighted averages were calculated to compare mice from different genotypes and treatments. Comparisons among subgroups (gender) were also conducted based on the model and the *P* values were adjusted using the Tukey–Kramer method. The histopathological features of the Zn supplemented and placebo groups were compared using Chi‐square tests. Statistical tests were two‐sided and were considered significant at *P *<* *0.05.

## Results

### *Fhit* ^−*/*−^ mice exhibit increased skin tumor burden

Previous studies showed that Fhit knockout mice are more sensitive than wt mice to carcinogen‐induced epithelial tumors of the forestomach and esophagus 14, 26. Therefore, we expected that *Fhit* ^−*/*−^ mice would also develop more NMSC tumors compared to wt B6 mice, a strain with low predisposition for spontaneous skin tumors (http://www.informatics.jax.org/mtbwi/tumorFrequencyGrid.do). Accordingly, each genotype included two treatment groups of mice, the control and Zn‐supplemented groups. Control mice received water without Zn, whereas the treatment group received Zn‐gluconate in drinking water, following initial DMBA treatment; 30 weeks after initial DMBA treatment, mice were sacrificed, skin tumors enumerated, and tissues fixed and processed.

The results, summarized in Table 2, demonstrate that unsupplemented *Fhit* ^−*/*−^ mice exhibited significantly more skin tumors following DMBA/PMA treatment than wt mice with an average of 16.2 versus 7.6 total tumors/mouse, respectively (*P *<* *0.001). Female and male *Fhit* ^−*/*−^ mice had similar multiplicities of total tumors (16.5/mouse for females and 15.9/mouse for males) whereas there was a slight bias for tumors in female wt mice (9.1/mouse for females vs. 6.0/mouse in males, *P *=* *0.07).

**Table 2:** Effect of Zn supplementation on skin tumor multiplicity in *Fhit* ^−*/*−^ and wt mice

Genotype	Treatment group	*n*	Total tumors/mouse	Number of tumors with size (Mean ± SD)		
			(Mean ± SD)	≤0.5 mm	1 mm	≥2 mm
B6 wt	F	23	9.1 ± 4.3	3.1 ± 2.6	4.1 ± 3.5	1.9 ± 1.6
	F + Zn	19	6.9 ± 4.5	2.2 ± 2.3	2.0 ± 1.6	2.7 ± 2.3
	M	11	6.0 ± 4.3	1.7 ± 1.6	2.4 ± 2.7	1.9 ± 1.4
	M + Zn	10	10.8 ± 5.4	6.8 ± 4.0	1.5 ± 1.3	2.5 ± 2.1
*Fhit* ^−*/*−^	F	28	16.5 ± 8.9	6.8 ± 4.7	6.1 ± 5.1	3.5 ± 2.5
	F + Zn	17	8.5 ± 6.3	3.2 ± 2.4	3.3 ± 3.7	2.0 ± 1.8
	M	15	15.9 ± 6.0	4.1 ± 3.6	7.4 ± 4.5	4.4 ± 3.6
	M + Zn	17	12 ± 4.6	4.3 ± 3.3	5.6 ± 4.0	2.1 ± 1.9

Statistical analyses showed statistically significant differences in tumor multiplicity (number of tumors per mouse, mean ± standard deviation) in unsupplemented *Fhit* ^−*/*−^ versus unsupplemented B6 mice (16.2 tumors in *Fhit* ^−*/*−^ mice versus 7.6 tumors in wt mice, weighted averages for genotypes combining males and females), *P *<* *0.001), as well as in tumor multiplicity in unsupplemented *Fhit* ^−*/*−^ mice versus Zn‐supplemented *Fhit* ^−*/*−^ mice (16.2 unsupplemented vs. 10.3 Zn supplemented, *P *=* *0.001).

F, female; M, male; B6, C57BL/6J.

### Skin tumor multiplicity in *Fhit* ^−*/*−^ mice was reduced by Zn supplementation

Zn supplementation significantly reduced total average and gender‐specific tumor burdens in *Fhit* ^−*/*−^ mice (see Table 2). *Fhit* ^−*/*−^ mice administered nonsupplemented drinking water developed more tumors (average, 16.2/mouse) compared to those on Zn‐gluconate water (10.3/mouse), a 37% decrease with Zn supplementation (*P *<* *0.001). Notably, the tumor burden reductions were different for female and male mice: female mice exhibited 49% reduced tumor burden after Zn treatment versus 25% for male mice. The tumor burden distribution, that is, fractions of tumors of ≤0.5, 1 and ≥2 mm diameter, was similar for male and female mice. In the *Fhit* ^−*/*−^ unsupplemented cohort, the distribution (% of tumors of various sizes) was 41%, 37%, 21% for females and 26%, 46%, and 28% for males. Within the *Fhit* ^−*/*−^ supplemented group, the distribution was 36%, 47%, and 16% for males and 37.6%, 39%, and 23% for females (smallest to largest).

On the other hand, Zn supplementation in wt mice did not have an observable effect on overall tumor number; wt mice without Zn supplementation averaged 7.6 tumors/mouse, whereas mice with Zn supplementation averaged 8.9 tumors/mouse (*P *=* *0.29). The tumor burden distribution among unsupplemented and supplemented wt mice shows more small tumors in the supplemented versus unsupplemented group: tumors of ≤0.5, 1 and ≥2 mm diameter were 63%, 14%, 23% and 32%, 29%, and 39%, respectively, for both males and females within the supplemented wt group and 28%, 40%, and 32% for males versus 34%, 45% and 21% for females of the Zn‐unsupplemented wt mice (Table 2). The greatest concentration of small tumors (≤0.5 mm) occurred in the supplemented wt male cohort, suggesting that tumors are continuing to be initiated in the supplemented wt males. This may suggest that the skin cancer‐resistant wt males exhibit not just a strain bias but also a gender bias, continuing to develop small tumors that with longer Zn treatment would likely regress.

We did observe a gender‐specific response to the Zn supplementation in wt females (9.1 tumors/mouse for no Zn versus 6.9 tumors/mouse for Zn supplemented, *P *=* *0.006), as seen in *Fhit* ^−*/*−^ females. While nonsupplemented wt males developed fewer tumors/mouse (6.0) than wt females (9.1), male mice developed more, mostly small tumors (10.8 total tumors per mouse) with Zn supplementation. The tumor size distribution in wt males clearly reflects the predominance of tumors of ≤0.5 mm in wt Zn‐supplemented mice versus those without Zn supplementation (6.8 vs. 1.7/mouse); since a previous study in oral carcinogenesis 15 showed that Zn supplementation caused a shift to a less aggressive tumor phenotype, we further analyzed the data excluding the tumors of ≤0.5 mm diameter. We then saw a decrease in the total number of large tumors (>1 mm) for both male and female wt mice following Zn supplementation: males, 4.3 reduced to 4.0; females, 6.0 reduced to 4.7, a decrease that is not statistically significant, likely due to the combined resistance of wt mice to skin tumors development, the small sample size and the fact that the tumor promotion protocol occurred in parallel with the Zn supplementation. It is highly likely that if the Zn supplementation of mice were continued beyond discontinuation of the tumor induction protocol, we would have seen a continuing regression of tumors in the supplemented mice versus unsupplemented cohorts.

### Histopathological analysis of skin lesions

Formalin‐fixed, paraffin‐embedded skin tumors and treated skin regions of all mice from each genotype were processed, embedded, mounted, and H&E stained for histopathological analyses. Several sections from each skin sample were scored for frequency of papilloma, mild or severe hyperplasia, dysplasia, and carcinoma. Results are summarized in Table 3 and sections illustrating tissues with hyperplasia, dysplasia, and SCC with different differentiation level are shown in Figure 1B. Skin SCCs were found in all the strain and treatment groups as listed in the final column of Table 3 and the incidence of SCCs was reduced in each cohort, male and female, by Zn supplementation: 35% and 80% of nonsupplemented female and male wt mice, respectively, showed SCCs in the skin sections examined and these were reduced to 22% and 20%, respectively, in the Zn‐supplemented females and males; in the *Fhit* ^−*/*−^ cohorts, 68% of females and 53% of males exhibited SCCs in the tissue sections examined and these SCC incidences were reduced to 58% and 29%, respectively. The most striking and significant reduction in SCC incidence was observed in the wt male cohort (80–20%, *P *=* *0.023); it is also very striking that in the untreated wt males, no papillomas were observed, as if all tumors progressed to SCCs, whereas the Zn treatment slowed this progression significantly, leaving more papillomas that have not progressed. Also, the overall SCC incidence (for both strains, males plus females) was reduced by Zn supplementation, achieving statistical significance in wt strain (*P *=* *0.035) and it is likely that continuation of Zn supplementation beyond termination of the 30‐week PMA tumor promotion treatments would have eliminated the skin SCCs entirely. Figure 1A illustrates the results of the 20‐week pilot tumor induction experiment illustrating the tumor initiation latency differences among the mouse strains and genders, showing that the B6 mice showed a longer latency period relative to the Fhit‐deficient mice.

**Table 3:** Histopathological analysis of skin lesions

Genotype	Treatment group	No. of mice examined	Incidence (%)				
			Papilloma	Mild hyperplasia	Severe hyperplasia	Dysplasia	SCC
B6 wt	F	23	4/23 (17%)	0	5/23 (22%)	6/23 (26%)	8/23 (35%)*
	F + Zn	18	5/18 (28%)	1/18 (6%)	3/18 (16%)	5/18 (28%)	4/18 (22%)
	M	10	0	0	0	2/10 (20%)	8/10 (80%)**
	M + Zn	10	4/10 (40%)	1/10 (10%)	0	3/10 (30%)	2/10 (20%)**
*Fhit* ^−*/*−^	F	28	3/28 (11%)	0	0	6/28 (21%)	19/28 (68%)*
	F + Zn	17	3/17 (18%)	1/17 (6%)	2/17 (12%)	1/17 (6%)	10/17 (58%)
	M	15	5/15 (34%)	0	0	2/15 (13%)	8/15 (53%)
	M + Zn	17	3/17 (18%)	0	4/17 (24%)	5/17 (29%)	5/17 (29%)

SCC incidence includes both carcinoma in situ and squamous cell carcinoma – *unsupplemented *Fhit* ^−*/*−^ female versus unsupplemented wt female (68% vs. 35%), *P *=* *0.026; **Zn supplementation led to a reduced carcinoma incidence in wt males (20% vs. 80%), *P *=* *0.023. Additionally, the total M+F wt SCC burden was significantly reduced in the mice with Zn supplementation (6/28 SCCs or 18% in Zn‐supplemented wt mice vs. 16/33 SCCs or 48% in unsupplemented wt mice, *P *=* *0.035); in Fhit^−/−^ mice, with more total M  +  F tumors in more mice, there was a decrease in SCCs with Zn supplementation, trending toward significance (15/34 SCCs or 44% with Zn supplementation vs. 27/43 or 63% SCCs without Zn supplementation, *P *=* *0.11). Fisher's exact test, two‐tailed. F, female; M, male; SCC, squamous cell carcinoma.

### The inflammatory process in skin of unsupplemented and Zn‐supplemented mice

**Figure 2:**
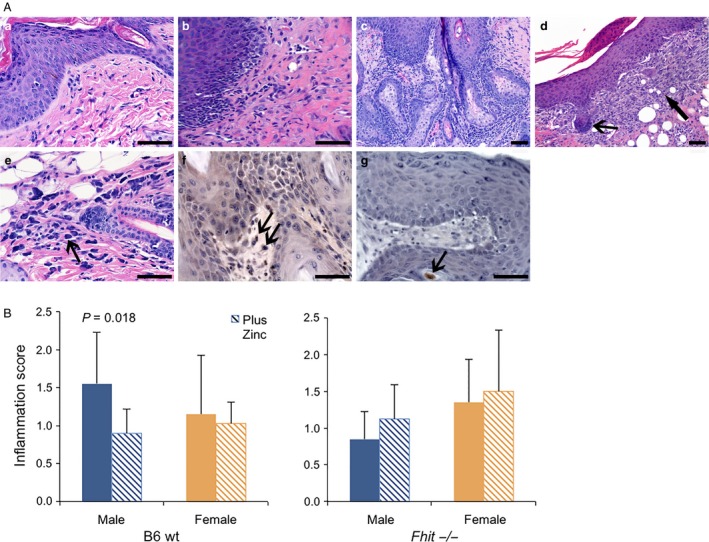
Inflammation in wt and *Fhit* ^−/−^ DMBA/PMA‐induced skin tumors. (A) H&E sections (a–e) showing (a) inflammation grade 0–1 with hyperplasia from a *Fhit* ^−*/*−^ female with Zn; (b) inflammation grade 2 with hyperplasia in skin epithelium from a *Fhit* ^−*/*−^ male with Zn; (c) inflammation grade 3 in hyperplasia from a *Fhit* ^−*/*−^ female without Zn supplementation; (d) inflammation in skin SCC in situ with marked fibroblast hyperplasia (thick arrow) and basal cell proliferation (thin arrow) from a wt female without Zn; (e) inflammation grade 0–1 with increased mast cells (mastocytosis, arrow) in skin with hyperplasia from a wt female without Zn; (f) showing antimyeloperoxidase immunostained section from a *Fhit* ^−*/*−^ female without Zn supplementation, with SCC and marked inflammation with increased mast cells, arrows point to immunostained positive mast cells; (*g*) antimyeloperoxidase immunostained severe hyperplasia from a *Fhit* ^−*/*−^ male with Zn treatment, showing reduced neutrophil and rare mast cell (arrow). Scale bar = 100 *μ*m. (B) Inflammation score in wt and *Fhit* ^−/−^ skin tumors from males and females with or without Zn supplementation (error bars = SD,* n* = 10–27/cohort).DMBA, dimethylbenzanthracene; PMA, phorbol myristate; SCC, squamous cell carcinoma.

Since inflammation is a contributing factor to carcinogenesis 28, we wanted to determine if Zn supplementation reduced tumor burden via attenuation of inflammation. We scored *Fhit* ^−*/*−^ and B6 for inflammation at the time of sacrifice on a scale of 0–3 (Fig. 2A). As shown in Figure 2B, B6 male skin tumors exhibited significant reduction in inflammation with Zn supplementation (from 1.55 ± 0.68 without Zn to 0.90 ± 0.32 with Zn supplementation, *P *=* *0.018), a result suggesting that Zn supplementation reduces SCC incidence in B6 males (Table 3) by lessening inflammation. *Fhit* ^−*/*−^ mice did not show statistically significant changes in inflammation scores after supplementation. This suggests that the observed reductions in tumor multiplicity via Zn supplementation in *Fhit* ^−*/*−^ mice involved mechanisms other than or in addition to effects on inflammatory processes.

### Expression of DNA damage response markers in skin tissues of Zn‐supplemented mice

**Figure 3:**
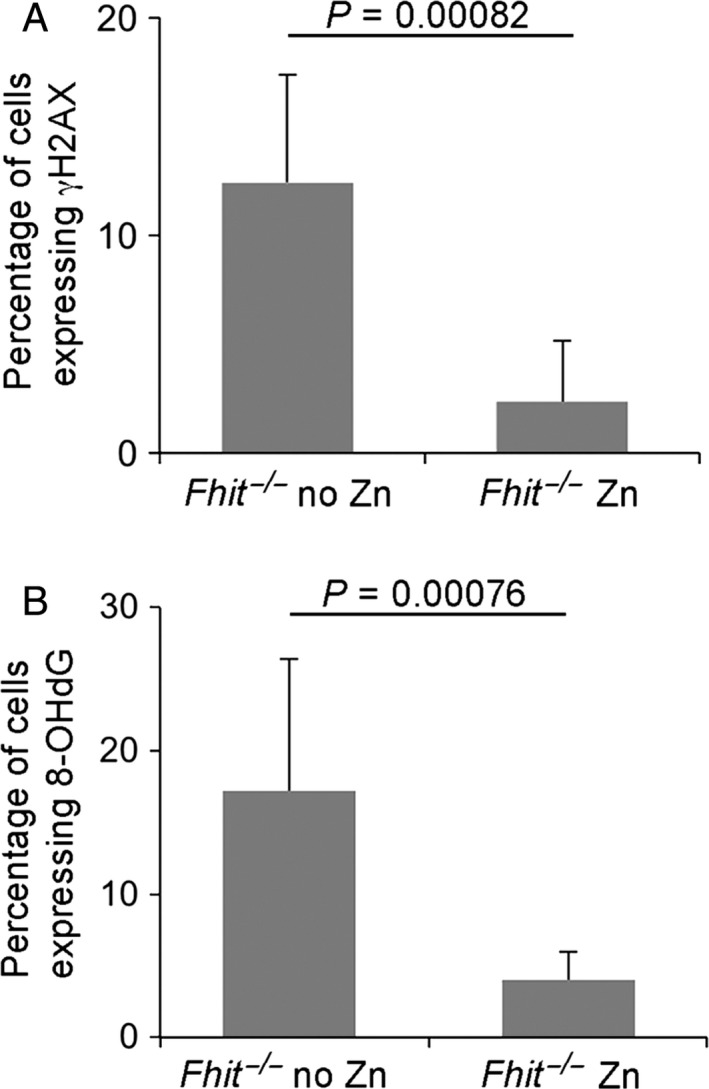
Zn supplementation reduces 8‐OHdG and *γ*H2AX expression in skin tumors in *Fhit* ^−*/*−^ mice. Bar charts showing percentage of (A) *γ*H2AX‐positive cells; (B) 8‐OHdG‐positive cells. The bar charts represent mean +/^−^SD. *n* = 7 for each treatment, compared with *Fhit* ^−/−^ without Zn, *P *=* *0.0008 for *γ*H2AX and *P *=* *0.0008 for 8‐OHdG.

Although the mechanism of DMBA/PMA‐induced tumorigenesis is not fully elucidated, it is thought to be partially through induction of DNA damage. To determine if the reduced level of skin lesions in Zn‐supplemented subjects was accompanied by changes in the level of expression of DNA damage markers, we assessed the presence of 8‐OHdG adducts and *γ*H2AX expression by IHC staining (Fig. 3). Focal *γ*H2AX expression is a marker of DNA damage at replication forks 29, whereas 8‐OHdG is an oxidized derivative of deoxyguanosine and is a major product of DNA oxidation 30. As shown in Figure 3, Zn supplementation significantly reduced 8‐OHdG accumulation (*P *=* *0.0008) and *γ*H2AX expression (*P *=* *0.0008) in lesions of *Fhit* ^−/−^ mouse skin, suggesting that Zn‐associated reduction in level of reactive oxygen species production and reduction in level of DNA damage contributed to the reduced tumor burden in skin of supplemented mice.

## Discussion

Dietary Zn is essential for proper physiological development and performance of numerous physiological functions including growth, reproduction, immune response, pregnancy, and prenatal development 9, 31, 32. Response to Zn ingestion exhibits a U‐shaped dose–response curve due to adverse effects associated with too little or too much Zn in vital tissues and organs. Rare cases of Zn toxicity, due to application of Zn‐based denture adhesives in one particular report, can lead to systemic accumulation of high levels of Zn, which in turn may cause pathologic copper‐deficiency and neurologic signs 33, these side effects were reversible on elimination of the source of Zn excess. However, the predominant area of research regarding Zn has been in deficiency, as it is estimated that approximately two billion people or one‐third of the world's population is affected by inadequate sources of dietary Zn 34. Geographically, this prevalence corresponds to regions in Africa, India, Southeast Asia, and Latin America 35. Studies have shown that Zn‐deficient populations receiving supplemental Zn have reductions in the incidences of acute lower respiratory infections, morbidity associated with diarrhea, and increased weight gain in infants and children 36. In addition, numerous epidemiological studies in humans, as well as experimental rodent models, have demonstrated that Zn‐deficiency contributes to cancer development 14, 37, 38.

We were interested in determining if mild Zn supplementation may contribute to reduction in skin cancer burden in Zn‐sufficient subjects, using a preclinical mouse model and carcinogen induction of skin cancer. We employed *Fhit* ^−*/*−^ mice because of their sensitivity to carcinogen induction of various tumor types, and B6 mice as wt control cohort. We have previously reported that Fhit deficiency causes genome instability that is undetected by cellular checkpoints 24, providing an optimal environment for selection and clonal expansion to survive various stressors 25. For instance, we have shown that *Fhit* ^−*/*−^ mouse kidney cells survive acute DMBA treatment and chronic severe nutritional stress whereas wt cells do not 25. Therefore, the enhanced survival and mild genomic instability characteristic of Fhit‐deficient mice and tissues allow them to serve as appropriate models for carcinogen‐induced cancer models. In line with our hypothesis, we found that *Fhit* ^−*/*−^ mice showed significantly more carcinogen‐induced tumors than wt mice (*P *<* *0.001). We expect that the skin of *Fhit* ^−*/*−^ mice treated with the DMBA/PMA induction protocol would also display increased single‐base substitutions, copy number alterations and aneuploidy, representative of the genome instability seen in *Fhit* knockout mice 25, 39, compared to the skin of the similarly treated control mice.

The data also confirmed our second hypothesis that *Fhit* ^−*/*−^ mice would exhibit statistically significant reductions in tumor burden with Zn supplementation following a DMBA/PMA induction protocol, suggesting a potential role for Zn supplementation in NMSC prevention. Interestingly, there was a gender bias in the effect of Zn supplementation in this model study, with supplemented female mice showing lower tumor burdens and thus appearing more sensitive to Zn supplementation compared to Zn‐supplemented males (49% reduction in females vs. 25% reduction in males). Our data agree with a growing body of evidence of a distinct gender bias in human and rodent models of skin cancer: biases that are not consistent across species. In humans, men exhibit a twofold higher incidence of NMSC than women. Sociological factors such as increased occupational sun exposure and less use of sun protective techniques among men are likely contributing factors. However, studies using experimental mouse models, where subjects are controlled for sun exposure and cancer monitoring, reveal significant biological differences between genders that contribute to the development of NMSC. For example, UVA radiation (320–400 nm) has an endogenous protective role by antagonizing UVB‐induced immunosuppression in a gender‐specific way, through the ER‐*β* pathway. That is, males, naturally deficient in estrogen and ER signaling, do not exhibit reductions in contact hypersensitivity induced by solar stimulated radiation, suggesting that females are more protected than males to NMSC. However, the same study demonstrated that male mice exposed to both UVA and UVB had less inflammatory edema compared to females due to their naturally thicker skin (presumably due to increasing thickness of the dermis and subdermal muscle layer) and decreases in levels of proinflammatory IL‐6 specific to males 40. In another study, Sullivan et al 41. proposed that lower endogenous catalase activity in males compared to females is responsible for increased NMSCs observed in male mice exposed to UVB radiation. Therefore, it is not surprising that we also see differences in the effects of Zn supplementation on NMSC in our model. Further studies will be needed to determine the causative factors for this gender disparity in response to DMBA and PMA initiation and promotion of carcinogenesis, though we show a clear association of tumor burden reductions with Zn supplementation in females for both genotypes.

Our observation that B6 male mice exhibited more tumors with Zn supplementation (10.8) than without (6.0), due to the increase in very small tumors (≤0.5 mm) in Zn‐supplemented B6 males (63%) versus unsupplemented males (28%), is also likely related to small sample size. Since it is thought that Zn supplementation works by suppressing the progression of neoplasia rather than inhibition of tumor initiation, the small tumors in the supplemented^−^/^−^ males may be due to persistence of small preneoplasias that are blocked in an early hyperplastic stage and cannot progress. In fact, if we remove the small tumors (≤0.5 mm) of this cohort from consideration, we observe a decrease in tumor burden with Zn supplementation.

The most important finding from this study, summarized in Table 3, is that each of the cohorts of mice, wt and Fhit‐deficient, male and female, showed reductions in the incidence of SCCs in the Zn‐supplemented groups, most significantly in the male wt mice, with a reduction of 80% of mice with SCCs without Zn supplementation to 20% with SCCs in the supplemented group, (*P *=* *0.023); interestingly these supplemented B6 male mice showed the highest number of small tumors and papillomas, as if these small tumors did not progress or regressed from SCCs. A larger study with Zn supplementation carried on for weeks after ending the PMA treatment would likely answer several of the questions raised by the results.

As a cofactor of over 300 mammalian proteins, dietary Zn has roles in a number of physiological processes 9, 32. Thus, we wanted to investigate mechanisms that might account for the significant reductions we observed in tumor burdens of Zn‐supplemented mice. First, we evaluated differences in inflammation grades in *Fhit* ^−/−^ mice Zn supplemented or not. Inflammation, particularly chronic inflammation, has been shown to predispose an individual to cancer 28. In fact, the widely used DMBA/PMA model of NMSC induction is based on chronic inflammation and the resultant production of inflammatory mediators 42. Zn is also known to help maintain the activity of cells involved in the immune response and inflammation, such as natural killer cells, neutrophils, and macrophage;^28^ therefore it was natural to presume that Zn supplementation may decrease tumor burdens by lessening the degree of inflammation caused by DMBA/PMA protocol. However, we did not observe a statistically significant difference in inflammation grades in *Fhit* ^−*/*−^ Zn‐supplemented and ‐unsupplemented groups. We then examined markers of DNA damage: 8‐OHdG adducts and *γ*H2AX expression by IHC staining in Zn‐supplemented and ‐unsupplemented groups, to determine if Zn lessened DNA damage through prevention of oxidative damage. We report a significant reduction in 8‐OHdG adducts, markers of DNA oxidation, and *γ*H2AX foci, markers of DNA double strand breaks, in supplemented *Fhit* ^−*/*−^ mice, suggesting that the method whereby Zn supplementation reduces tumor burden in *Fhit* ^−*/*−^ mice is by attenuation of oxidative damage. The precise antioxidant effect of Zn remains unclear, although two potential mechanisms exist: (1) Zn is an essential component of CuZnSOD, an enzyme that removes the superoxide anion in reactive oxygen species, and (2) Zn prevents the oxidation of sulfhydryl groups in redox‐active transition metals 43. Further studies are needed to determine if either or both of these mechanisms are responsible for the reductions in 8‐OHdG adducts and *γ*H2AX foci, but the data does suggest that Zn supplementation decreases tumor burden through DNA damage prevention, allowing for the identification of an anti‐inflammatory therapeutic as a possible synergistic regimen to Zn supplementation.

The results of this study suggest that mild Zn supplementation may be helpful for preventing the progression of NMSCs, even in Zn‐sufficient individuals, possibly in combination with other preventive treatments currently under investigation in UV or DMBA/PMA induction models, some showing promise 44, 45, 46, 47, 48, 49. Further studies are needed to define mechanisms that enable dietary Zn supplementation to reduce tumor burdens in *Fhit* ^−*/*−^ mice, perhaps with a focus on experiments aimed at evaluating differences in responses in male and female mice, since females displayed the most emphatic reductions. We believe that in clinical prevention or therapeutic trials in humans, particularly in cohorts predisposed to multiple skin cancers, it would be beneficial to include very inexpensive Zn supplementation in trial arms, to confirm its role in prevention of NMSC progression.

## Conflict of Interest

None declared.
